# New Insights into Mutable Collagenous Tissue: Correlations between the Microstructure and Mechanical State of a Sea-Urchin Ligament

**DOI:** 10.1371/journal.pone.0024822

**Published:** 2011-09-14

**Authors:** Ana R. Ribeiro, Alice Barbaglio, Cristiano D. Benedetto, Cristina C. Ribeiro, Iain C. Wilkie, Maria D. C. Carnevali, Mário A. Barbosa

**Affiliations:** 1 INEB- Instituto de Engenharia Biomédica, Biomaterials Division, NEWTherapies Group, Universidade do Porto, Porto, Portugal; 2 FEUP- Faculdade de Engenharia da Universidade do Porto, Departamento de Engenharia Metalúrgica e de Materiais, Porto, Portugal; 3 Biology Department, University of Milan, Milano, Italy; 4 ISEP- Instituto Superior de Engenharia do Porto, Departamento de Física, Porto, Portugal; 5 Department of Biological and Biomedical Sciences, Glasgow Caledonian University, Glasgow, Scotland; 6 ICBAS- Instituto de Ciências Biomédicas Abel Salazar, Universidade do Porto, Porto, Portugal; University of Crete, Greece

## Abstract

The mutable collagenous tissue (MCT) of echinoderms has the ability to undergo rapid and reversible changes in passive mechanical properties that are initiated and modulated by the nervous system. Since the mechanism of MCT mutability is poorly understood, the aim of this work was to provide a detailed morphological analysis of a typical mutable collagenous structure in its different mechanical states. The model studied was the compass depressor ligament (CDL) of a sea urchin (*Paracentrotus lividus*), which was characterized in different functional states mimicking MCT mutability. Transmission electron microscopy, histochemistry, cryo-scanning electron microscopy, focused ion beam/scanning electron microscopy, and field emission gun-environmental scanning electron microscopy were used to visualize CDLs at the micro- and nano-scales. This investigation has revealed previously unreported differences in both extracellular and cellular constituents, expanding the current knowledge of the relationship between the organization of the CDL and its mechanical state. Scanning electron microscopies in particular provided a three-dimensional overview of CDL architecture at the micro- and nano-scales, and clarified the micro-organization of the ECM components that are involved in mutability. Further evidence that the juxtaligamental cells are the effectors of these changes in mechanical properties was provided by a correlation between their cytology and the tensile state of the CDLs.

## Introduction

The ‘mutable’ collagenous tissue (MCT) of echinoderms (starfish, sea-urchins and their relations) has the capacity to undergo reversible changes in mechanical properties (viscosity, tensile strength, and stiffness) within timescales of around 1 s that are under the control of the nervous system [1], [2]. MCT is present in all living echinoderm classes, in the form of dermal connective tissue, interossicular ligaments and tendons [2]. In addition to fulfilling the mechanical functions associated with ‘conventional’ collagenous structures (i.e. energy storage, transmission and dissipation), MCT provides mechanisms for the detachment of appendages or body parts in response to disease, trauma or predator attack [2] and for the energy-sparing maintenance of posture [3]. Most mutable collagenous structures consist largely of parallel aggregations of collagen fibrils to which proteoglycans are covalently and non-covalently attached, as in mammalian connective tissue [2]–[11]. An elastomeric network of microfibrils surrounds and separates collagen fibers (bundles of fibrils), maintaining their organization and providing a long-range restoring force [2], [12], [13]. One constant morphological feature appearing in all MCTs is the presence of juxtaligamental cells (JLCs), which contain large electron-dense granules and come into close contact with motor axons [2], [14], [15].

The mechanical adaptability of MCT depends on the modulation of interfibrillar cohesion, and there is good evidence that this is mediated by effector molecules secreted from the JLCs [2]. Candidate effector molecules that influence the mutability of sea-cucumber dermis have been isolated and partly characterized [16]–[19].

The aim of our investigation was to advance knowledge of the basic biology of MCT by (i) providing a detailed morphological analysis of a typical mutable collagenous structure - the compass depressor ligament (CDL) of a sea-urchin, and (ii) identifying changes in morphological aspects of its cellular and extracellular components that are correlated with different mechanical states (‘standard’, ‘stiff’ and ‘compliant’). Using histochemistry, transmission electron microscopy (TEM), cryo-scanning electron microscopy (CSEM), focused ion beam/scanning electron microscopy (FIB/SEM), and field emission gun-environmental scanning electron microscopy (FEG/ESEM), we detected differences in both extracellular and cellular constituents of CDLs in different mechanical states, which provide an insight into the micro-organizational basis and control of MCT mutability. Our results help to characterize the functional role of this specialized ECM, which has striking morphological similarities to mammalian collagenous ECM.

## Materials and Methods

### Experimental animals and solutions

Sea-urchins (*Paracentrotus lividus*) for the TEM and FIB/SEM analyses were collected by scuba divers along the Ligurian coast of Italy and kept in tanks of aerated seawater at 16°C in the University of Milan. Animals for the FEG/ESEM, CSEM and histochemical analyses were collected in Aguda on the northern Portuguese coast and kept in tanks of seawater at 16°C in Estação Litoral da Aguda.

**Figure 1:**
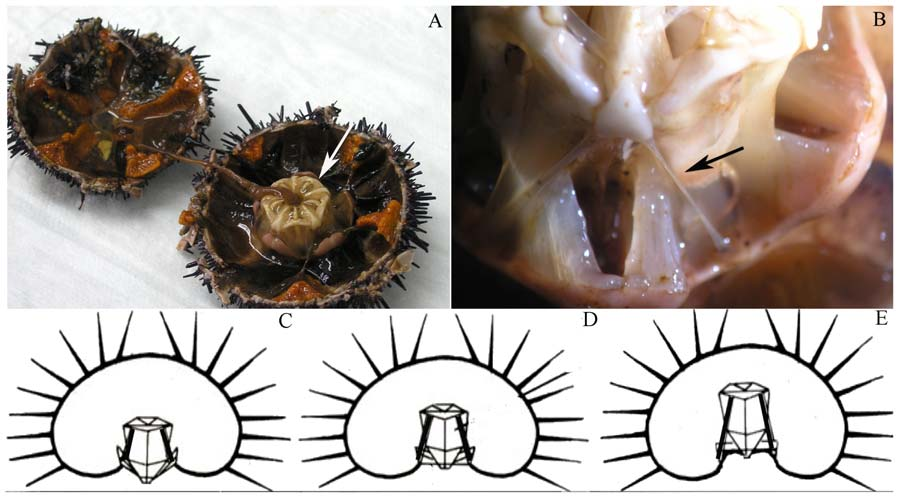
Anatomical relations and behavior of CDLs. (A, B) Specimen of *P. lividus* split into two halves: (A) The Aristotle's lantern (arrow) is observable in the oral half. (B) Enlargement of the Aristotle's lantern showing the anatomical location of the CDLs (arrow). (C–E) Diagrams of sea-urchins with lanterns in different positions; CDLs shown in black. (C) Protracted position; CDLs compliant; (D) Resting position; CDLs in standard state; (E) Retracted position; CDLs stiff.

CDLs are components of the masticatory apparatus (‘Aristotle's lantern’) of the sea-urchin (Fig. 1A). Each lantern contains ten CDLs (Fig. 1B) whose functions include stabilizing the position of the lantern [20], [21], [22]. In order to compare CDL morphology in different functional states, animals were subjected to three different treatments. To obtain the ‘compliant’ condition, the lower half of an animal, which includes the lantern, peristomial membrane and CDLs (Fig. 1A), was immersed in an anesthetic solution of 0.1% propylene phenoxetol (Sigma Aldrich 484423) in seawater for 45 min [20], [23]; this treatment results in the protraction (lowering) of the lantern and slackening of the CDLs (Fig. 1C). To obtain the ‘stiff’ condition [21], [22], half animals were immersed in 1 mM acetylcholine chloride (Sigma Aldrich 6625) in seawater for 15 min, which causes retraction (raising) of the lantern and stretching of the CDLs (Fig. 1E). Controls, which were in the ‘standard’ condition’ (Fig. 1D), were kept in seawater alone [21]. We chose to induce mechanical changes in intact CDLs left *in situ* in the lantern, rather than use excised CDLs held artificially at a constant length, in order to mirror as closely as possible *in vivo* conditions, where CDLs tend to undergo simultaneous changes in stiffness and length.

### Ethical treatment of animals

This study was carried out where no specific permits were required for the described field studies since sea-urchins (Paracentrotus Lividus) are invertebrates. This work was performed with a species that is not endangered or protected. The location of the field studies is also not privately owned or protected in any way.

### TEM and LM

After incubation in the respective solutions, half animals were fixed at 4°C for 2 h with 2% glutaraldehyde in 0.1 M cacodylate buffer. After an overnight wash in the same buffer, the specimens were post-fixed for 2 h with 1% osmic acid in 0.1 M cacodylate buffer. After fixation specimens were washed with distilled water. CDLs were removed carefully from the lantern, pre-stained with 2% uranyl acetate in 25% ethanol for 2 h, dehydrated in a graded ethanol series and embedded in an Epon-Araldite resin mixture. Semi-thin (0.90–0.99 µm) and thin (0.1–0.075 µm) sections were cut with an LKB V Ultrotome using a diamond knife. Thin sections were stained with aqueous uranyl acetate and lead citrate, and observed in a JEOL 100SX TEM. Semi-thin sections were stained with basic fuchsin and crystal violet and observed in a Jenaval light microscope.

### FIB/SEM

FIB/SEM samples were treated as above as far as the dehydration step, after which the ethanol was progressively substituted with hexamethyldisilazane (HMDS) and the samples allowed to dry at room temperature. They were then mounted on a stub, carbon coated and observed in a Dual Beam system for FIB/SEM operation (FEI Strata DB 235 M). Milling was performed in the form of a rectangle block with different dimensions (20×40 µm or 20×15 µm), with ion currents ranging from 10 pA to 30 nA, and beam energy of 30–40 keV. Lower beam currents were used for the cleaning mill. SEM imaging was obtained by means of the electron column available. The system operated with column pressures in the 10^−5^ Pa range and the specimen chamber pressure between 10^−4^ and 10^−3^ Pa.

### FEG/ESEM

Two sample procedures were followed. In the first the lantern complex was processed as for the FIB/SEM samples. In the second, samples were processed and embedded as for TEM and the internal surface of the resin-embedded CDLs was exposed by cutting a few longitudinal semithin sections. The resin blocks containing CDLs were placed in contact with a solution of 1% NaOH in absolute ethanol at room temperature for 30 minutes to dissolve the resin and expose the internal structure of the tissue for observation with FEG/ESEM. After resin dissolution, CDLs were washed in 100% ethanol and dried in HMDS. All CDLs were then observed in a FEI Quanta 400FEG ESEM/EDAX Genesis X4M microscope at low vacuum (70 Pa) without any coating.

### CSEM

Unfixed and hydrated CDLs were examined by CSEM. Immediately after dissection, CDLs were immersed in liquid nitrogen and carefully mounted onto a cryo sample holder. By means of a vacuum cryo transfer system, CDLs were placed on the cryo-stage where they were kept under high vacuum at a minimum temperature of −210°C. After appropriate positioning of the stage, a blade was used to create a transverse freeze fracture. Water was removed by gentle sublimation at −90°C for 5 min. The CDLs were sputter-coated with gold palladium for 80 s and the fracture face was inspected in a JEOL JSM 6301F/Oxford INCA Energy 350/Gatan Alto 2500.

### Proteoglycan histochemistry

#### Alcian blue

CDLs were fixed with 4% paraformaldehyde in PBS, dehydrated in an ethanol series, cleared in xylene and embedded in paraffin wax. Sections 7 µm thick were cut in a Reichert Jung microtome, deparaffinized with xylene and hydrated to distilled water. Sections were stained with alcian blue 8GX (Sigma-Aldrich 05500; staining solution: 1% alcian blue in 3% acetic acid, pH 2.5) for 30 min, washed in running tap water for 2 min, rinsed in distilled water, dehydrated in an ethanol series, and washed in xylene. The sections were observed in a Jenaval light microscope.

#### Cuprolinic blue

Half animals in the standard state were fixed overnight at room temperature in 0.1% cuprolinic blue containing 2.5% glutaraldehyde in sodium acetate 0.025 M and MgCl_2_ 0.3 M (pH 5.6). CDLs were washed twice in sodium acetate 0.025 M and MgCl_2_ 0.3 M (pH 5.6) followed by a wash in distilled water. After fixation, CDLs were removed carefully from the lantern under distilled water, washed in ethanol 25%, and in 0.5% sodium tungstate in ethanol 50%, dehydrated in a graded ethanol series and embedded in an Epon-Araldite resin. Sections (70–100 nm) were cut with an LKB V Ultrotome using a diamond knife, stained with aqueous 2% uranyl acetate in distilled water, and observed in a JEOL 100SX TEM.

### Quantitative evaluation and statistical analysis

TEM micrographs at a magnification of ×30,000 were acquired of one CDL from each of four different animals. Twenty-five measurements of the distance between adjacent collagen fibrils were obtained in each micrograph, using the program ImageJ 1.41o. Since the data were not normally distributed (D'Agostino and Pearson test), the Mann–Whitney U test was used to compare the distance between collagen fibrils in standard, stiff and compliant CDLs. Results were considered statistically significant when p<0.05. ImageJ 1.41o was also used to measure the diameter and number of the ‘dark’ (i.e. completely electron-opaque) and ‘light’ (i.e. partly electron-lucent) granules inside JLCs in TEM images (×16,000). These cytological data were subjected to statistical analysis, and results were considered statistically significant when p<0.05. Means are given ± standard deviation. Statistical differences between CDLs in different functional states were determined using Kruskal-Wallis one-way analysis of variance (ANOVA) with Dunn's post-hoc test. All statistics were performed using GraphPad Prism 5 Demo software (version 5.02).

## Results

### Background: microstructural organization of the CDL

**Figure 2:**
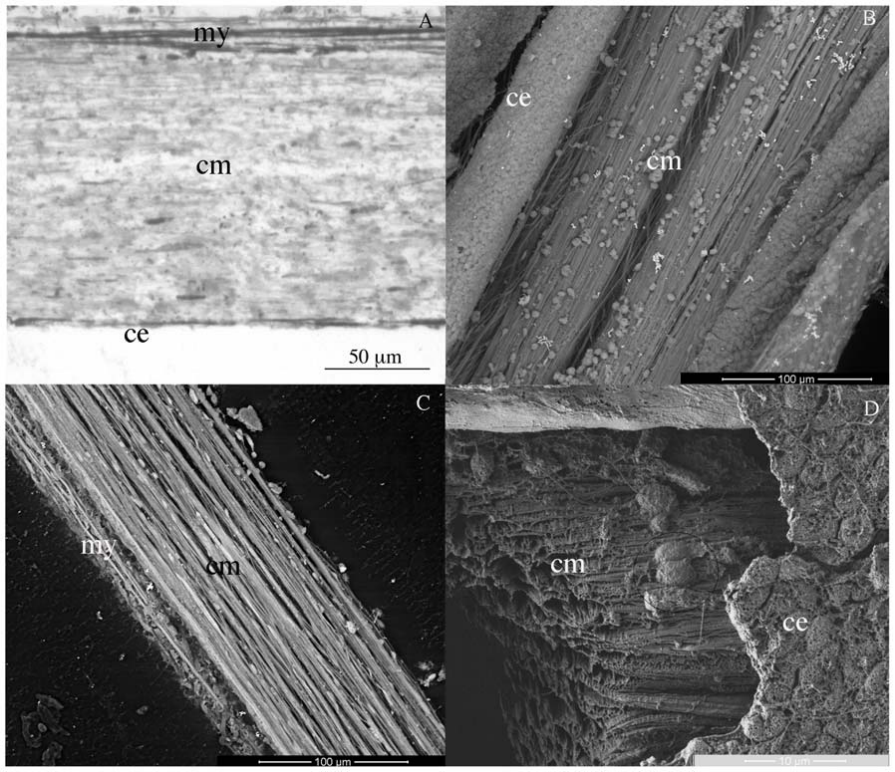
General view of CDL internal structure. (A) Semi-thin longitudinal section of CDL and (B, C) FEG/ESEM micrograph of resin-embedded CDL revealing the dense collagen array surrounded by a coelomic epithelium. Fig.2A shows clearly the coelomic myoepithelium. (D) FIB/SEM micrograph of milled CDL showing small globular cells between the collagen bundles covered by a ciliated coelomic epithelium. ce, coelomic epithelium; my, myoepithelium; cm, collagen matrix.

Each CDL was a strap-shaped band of tissue 9–10 mm long and 0.2–0.4 mm wide, and consisted mainly of a parallel aggregation of cross-striated collagen fibrils. The fibrils were enclosed within a coelomic epithelium, which became a myoepithelium on one side of the CDL (Fig. 2A). The parallel organization of the collagen fibrils with globular cells was shown well by FEG/ESEM and FIB/SEM (Fig. 2 B, C, D).

**Figure 3:**
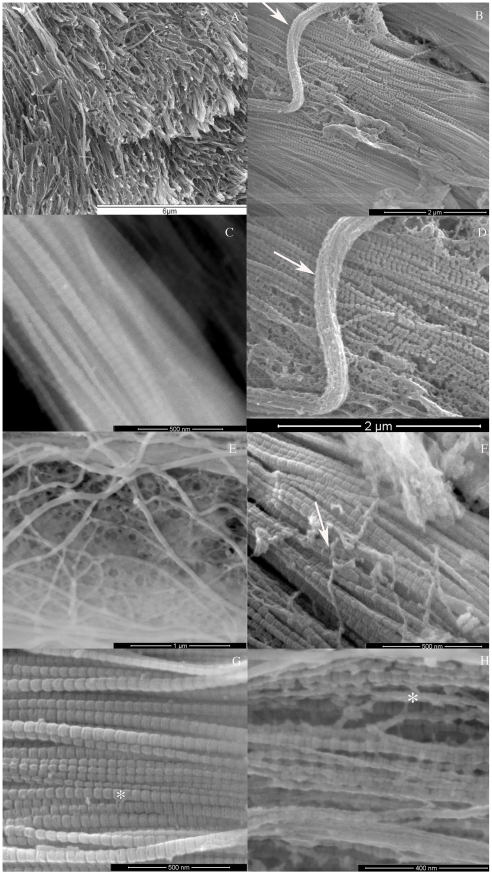
CDL extracellular matrix. (A) Cryo-SEM and (B–H) FEG/ESEM micrographs showing a (A) general view of CDL compliant matrix. (B) Detail of collagen fibrils and fibrillin (arrow). (C) Parallel array of collagen fibrils showing a clear D-banding pattern. (D) Enlargement of Fig. 3B showing a fibrillin bundle (arrow). (E) Enlargement of Fig. 3B showing a fibrillin meshwork. (F) Loose fibrillin meshwork (arrow) on surface of collagen fibrils. (G, H) Interfibrillar bridges linking adjacent fibrils (*).

Fig. 3A, B, C shows that the fibril diameter was variable, and measurements of TEM images revealed that fibril diameters ranged from 26.52 nm to 179.42 nm (mode 45.5±19.0 nm; N = 1033). The nanometer resolution of TEM revealed that the D-spacing of the cylindrical collagen fibrils (Fig. 3 C, F) varied from 39.7 to 77.9 nm (mean 59.2±6.2 nm; N = 344). The collagen fibrils were aggregated into bundles (i.e. ‘fibers’) with a wide range of diameters (0.4–1.3 µm; mean 0.87±0.46 nm; N = 13). Between the collagen fibers there were beaded filaments of mean diameter 25.6±10.3 nm (N = 24), which formed a loose meshwork or compact bundles with mean diameter 245±16.5 nm (N = 14) (Fig. 3B, D–F). In addition to these elongated filaments, short filamentous structures of variable length (10–30 nm) were observed by FEG/ESEM to directly connect adjacent collagen fibrils, thus forming interfibrillar bridges (Fig. 3G, H).

**Figure 4:**
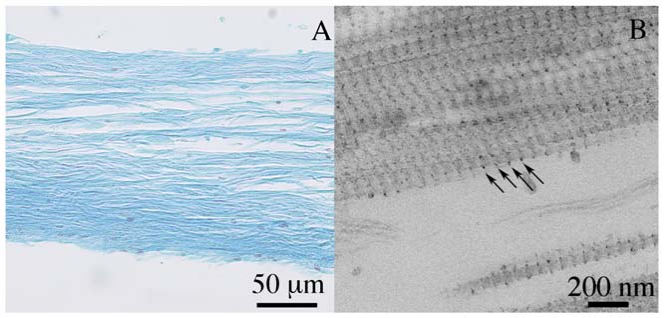
Proteoglycans presence and distribution. Longitudinal section of CDL stained with (A) alcian blue at pH 2.5 and cuprolinic blue (B) (arrows, cuprolinic blue stained precipitates).

The fibres of the CDL were stained moderately and uniformly by alcian blue at pH 2.5, indicating the presence of GAGs (Fig. 4A). Staining of CDLs with cuprolinic blue produced electron-dense globular or ellipsoid precipitates on the surface of the collagen fibrils. On each side of a particular collagen fibril, one such precipitate was located within each D-period and at the same location within each D-period, so that the precipitates were spaced very regularly (Fig. 4B).

**Figure 5:**
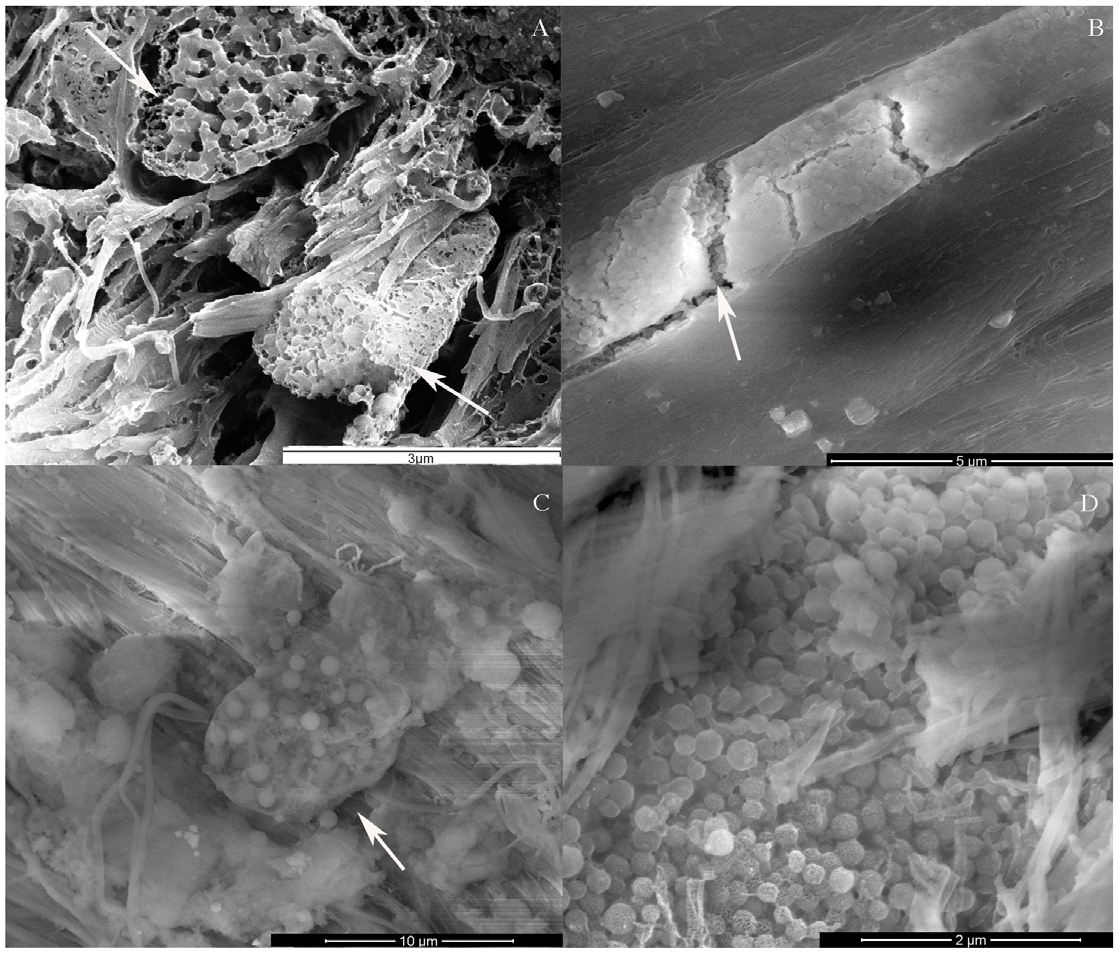
Juxtaligamental cells. (A) CSEM micrograph showing a transverse section of a JLC (arrow) within the collagen array. (B–D) FEG/ESEM micrographs of resin- embedded samples. The dissolution of resin allows the direct visualization of a fractured JLC process (arrow), showing (B) the internal granules, (C) granules inside the cell membrane (arrow), and (D) the variation in JLC granule size.

The ligament also contained many granule-containing cell bodies and processes (Fig. 5), which will be described below.

### Microstructural organization of CDLs in the different mechanical states

#### Gross changes

Anesthetization reduced the stiffness of all MCT in the lantern, including that of the peristomial membrane to which the lantern is attached. As a result, the entire masticatory apparatus was protracted and the CDLs shortened and thickened (Fig. 1 C). In response to acetylcholine, the CDLs and peristomial membrane became stiff, the lantern was helded in a retracted position, and the CDLs were longer and thinner than in the standard condition (Fig. 1 E).

#### Microstructural changes

**Figure 6:**
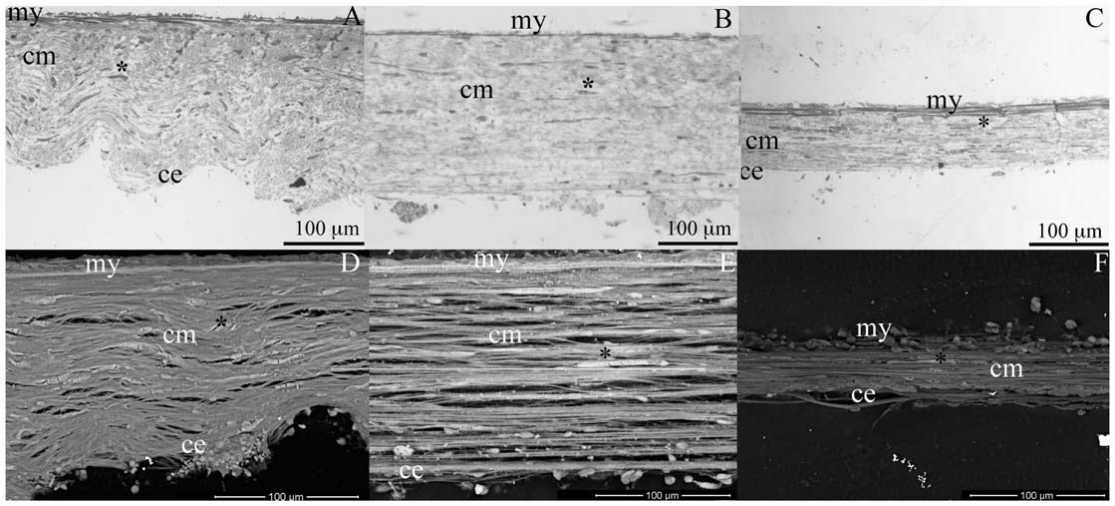
Histology of CDLs in different functional states. (A–C) Semi-thin longitudinal sections and (D–F) FEG/ESEM micrographs showing CDLs in the compliant state (A, D), standard state (B, E) and stiff state (C, F). Asterisks, cells; ce, coelomic epithelium; cm, collagen fibers; my, myoepithelium.

Histological sections and FEG/ESEM micrographs showed that the organization of the collagen array and cells changed when CDLs shift from the standard to the stiff or compliant states (Fig. 6).

#### Extracellular components

**Figure 7:**
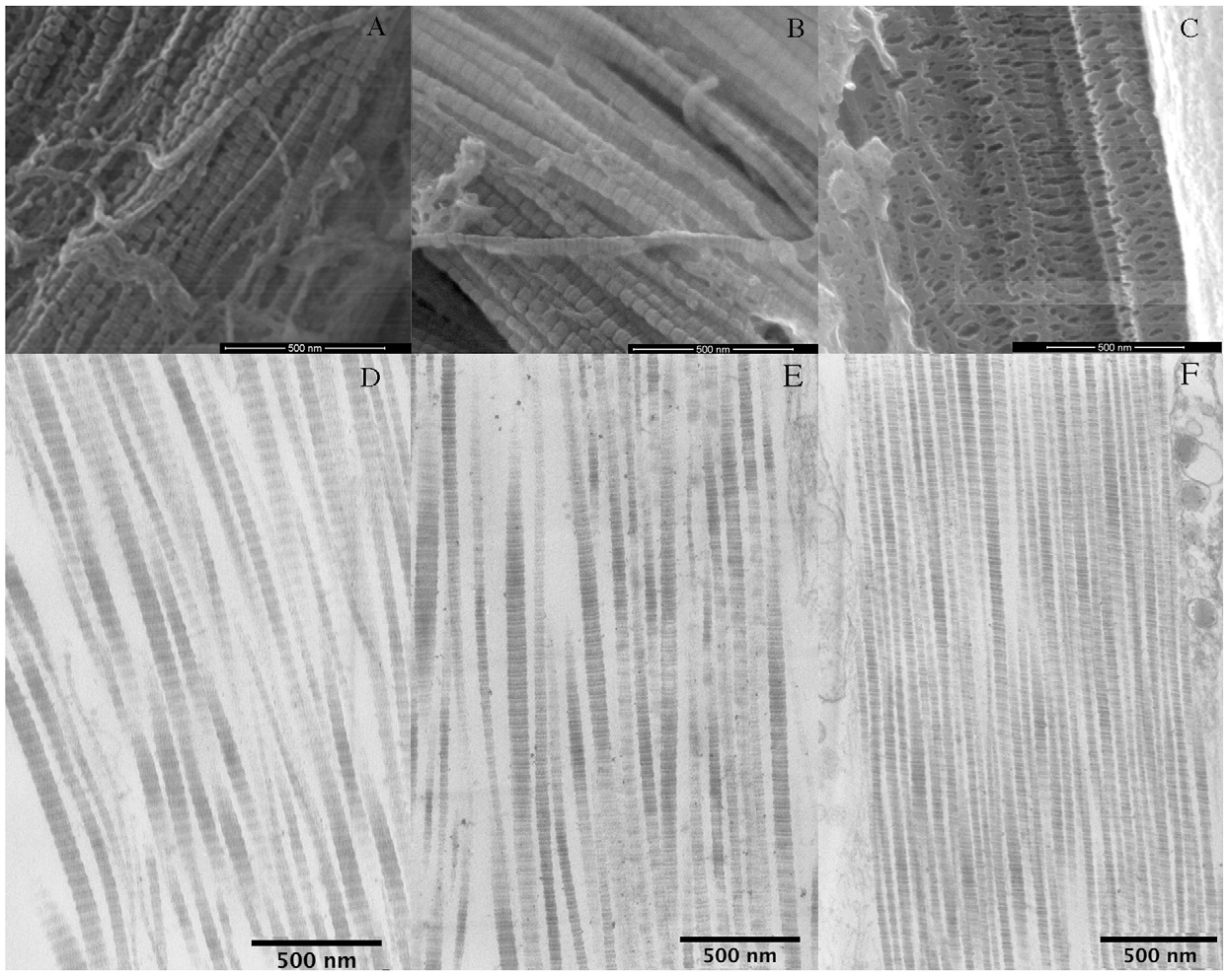
Arrangement of collagen fibrils. (A–C) FEG/ESEM and (D–F) TEM micrographs. (A, D) Compliant state. (B, E) Standard state. (C, F) Stiff state.

**Figure 8:**
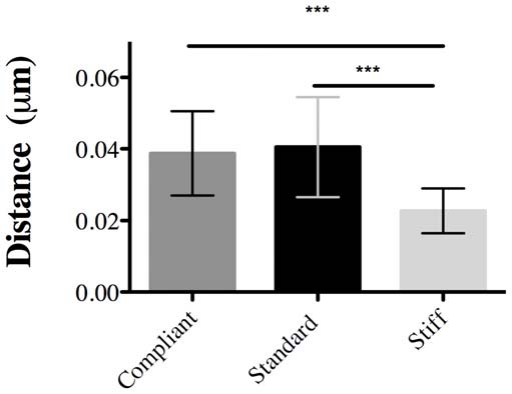
Distance between collagen fibrils. Mean interfibrillar distance of CDLs in the three mechanical states. ***P<0.0001. Data are expressed as means ± SD.

Collagen fibrils were more tightly packed in stiff CDLs than in compliant or standard CDLs (Fig. 7). The mean interfibrillar distance was significantly lower in stiff CDLs than in compliant and standard CDLs (Mann Whitney U: 5446, P<0.0001 and U: 7617, P<0.0001 respectively), although there was no significant difference between compliant and standard CDLs (Fig. 8).

**Figure 9:**
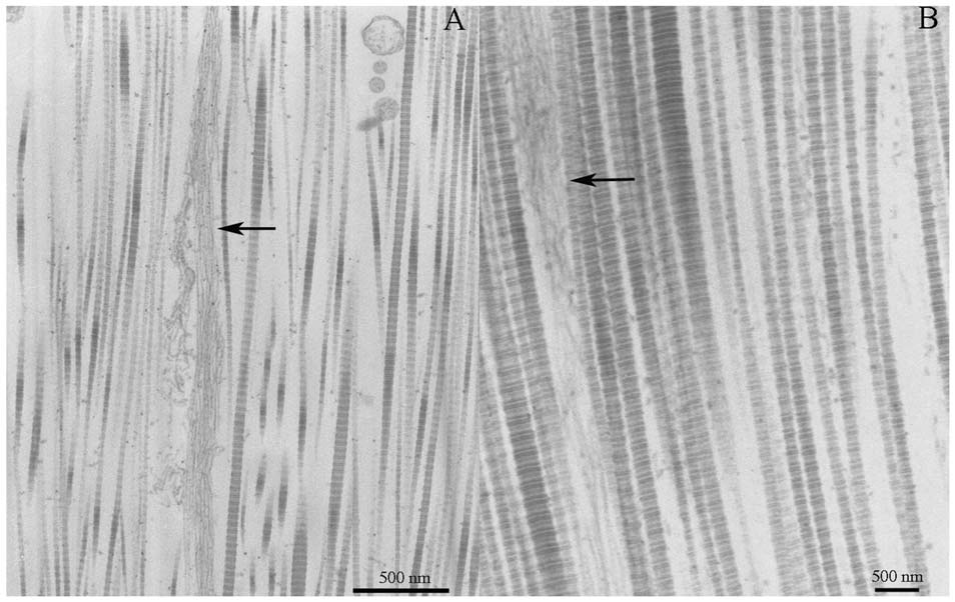
Microfibrils. TEM micrographs showing the distribution of microfibrils in CDLs in standard (A) and stiff (B) states.

The only effect of mechanical state on the distribution of fibrillin microfibrils seemed to be a consequence of collagen fibril packing. In the stiff condition, the reduction in interfibrillar space resulted in the microfibrils forming more densely packed sheets (Fig. 9B). In the compliant and standard conditions they are more dispersed between the collagen fibrils (Fig. 9A).

#### Cellular components: JLCs and intracellular granules

**Figure 10:**
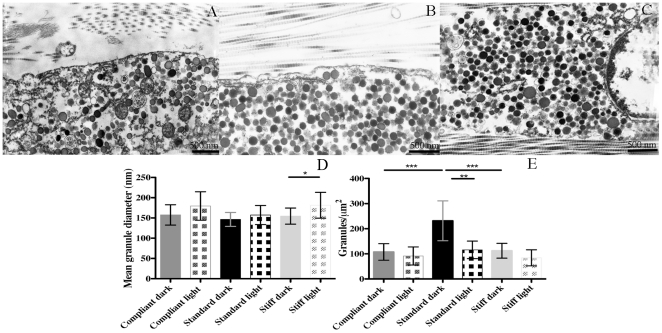
JLCs in the different mechanical states. (A–C) TEM micrographs of compliant (A), standard (B) and stiff (C) CDLs. (D) Size of JLC granules in different mechanical states. *P<0.05. (E) Number of JLC granules in different mechanical states. **P<0.01; ***P<0.001. Data are expressed as means ± SD.

The main cellular elements observed in TEM and FEG/ESEM contained many membrane-bounded, electron-dense granules, and thus resembled the juxtaligamental cells (JLCs) that are present in all previously examined mutable collagenous structures in echinoderms. The profile of the granules was usually circular. The presence of less frequent oval profiles suggested that at least some of the granules were ovoid rather than spheroidal in shape (Fig. 10A–C). Fig. 10D showed that in all three mechanical states, the mean diameter of ‘dark’ (completely electron-opaque) granules was lower than that of ‘light’ (partly electron-lucent) granules, although the difference was statistically significant only for stiff CDLs. The size of neither the dark granules nor the light granules differed significantly between the different mechanical states. Regarding granule abundance, dark granules were more numerous than light granules in all three mechanical states, although the difference was significant only in the standard condition. Dark granules were considerably less numerous in compliant and stiff CDLs than in standard CDLs, whereas there was no significant difference between the numbers of light granules in the three mechanical states (Fig. 10E).

## Discussion

### Organization of CDL microenvironment

The CDL of *P. lividus* resembles most other echinoderm mutable collagenous structures in consisting mainly of collagen fibrils with a banding periodicity of ca. 60 nm [21]. Available data on the molecular biology of collagens from different MCTs indicate that they are evolutionarily close to those of vertebrate fibrillar collagens and share no features that can be correlated with the mechanical adaptability of the tissues [2], [24]–[27]. In the CDL, the collagen fibrils are organized into parallel fibers that determine the tensile strength of the ligament and set a limit on the degree to which it can stretch during routine movements of the sea-urchin's lantern [20]. It is interesting to note that the general architecture of CDL collagen, such as fibril organization and bundle orientation, is identical to the structure and organization of collagen observed in several human tissues such as tendon, ligament, cornea, skin and blood vessels [28].

Beaded filaments like those forming a meshwork or bundles between the collagen fibers of the CDL are ubiquitous in MCT and are also present in echinoderm ligaments that show no evidence of mutability [2]. Those in the mutable dermis of a sea-cucumber are morphologically, biochemically and immunologically similar to mammalian fibrillin-containing microfibrils [13], [29], and a preliminary investigation has detected fibrillin-1-like immunoreactivity in the CDL of *P. lividus* (Barbaglio, unpublished results). In vertebrates and invertebrates, aggregations of fibrillin-rich microfibrils are thought to provide tissues with the capacity for strain energy storage and elastic recoil [29], and we envisage that shortening and thickening of CDLs, which we observed in intact preparations of the lantern treated with propylene phenoxetol, is due in part to microfibrillar mechanisms.

The CDLs were stained by alcian blue at pH 2.5, which is fairly selective for GAGs [5], [30]. GAGs, and the proteoglycans (PGs) into which they are incorporated as side-chains, are likely to play a significant role in the mechanical adaptability of MCT, since they are directly or indirectly involved in interfibrillar cohesion, the reversible modulation of which underpins the mutability phenomenon [2], [31]. PGs were visualized by the polycationic dye cuprolinic blue, demonstrating that polyanions are periodically distributed along collagen fibrils surface, being attached to specific sites in each D-period of the collagen fibril. Scott [32] demonstrated that collagen fibrils in holothurian dermis are surrounded and interconnected by a PG lattice of orthogonal and axial filaments, and interfibrillar PGs have been identified in a sea-urchin spine ligament [7], [31], [33], [34]. The short interfibrillar cross-bridges observed by FEG/ESEM in the CDL may have been PGs, although they were not periodically distributed.

### Correlations between microstructure and mechanical state

This investigation has revealed previously unreported differences in both extracellular and cellular constituents of a representative mutable collagenous structure, the sea-urchin CDL, in different mechanical states that mimic the mutability of the tissue *in vivo*. We found that the mean interfibrillar distance of stiff CDLs was significantly lower than that of standard or compliant CDLs, and that there was no significant difference between those in the standard and compliant state. The denser fibril packing of the stiff CDLs obviously involves a reduction in interfibrillar space and displacement of materials previously occupying that space. This is probably a result of the stretching of the CDLs in acetylcholine-stimulated preparations and, by shortening the distance between adjacent fibrils, may facilitate the stiffening mechanism by making it easier for interfibrillar cohesion to be strengthened, for example by the attachment of crosslinking agents such as tensilin [16]. Such facilitation of the stiffening mechanism may occur in other mutable collagenous structures that consist of parallel fibril arrays and undergo simultaneous stretch and stiffening, such as sea-urchin spine ligaments (“catch apparatus”) [35], crinoid arm ligaments [36] and ophiuroid arm ligaments [37].

Like all previously examined mutable collagenous structures, the CDL contains many juxtaligamental cell (JLC) bodies and their processes. These cells, characterized by their abundant intracellular granules, are probably the effector cells that directly bring about changes in the tensile properties of the extracellular matrix, because (1) they terminate within MCT, (2) they are in close contact with the motor nervous system, (3) immunological methods have demonstrated the presence in their granules of molecules that influence MCT tensility (such as tensilin and stiparin), and (4) there is no other candidate cell-type within or near MCT [2], [17]–[19], [38]. The electron density of the granules in the JLCs of the CDL is variable, as has been observed in all other MCTs, and for the purposes of this investigation the granules were categorized as being ‘dark’ or ‘light’. We suspect that these represent only different stages in granule maturation, the dark being fully mature and the light various immature stages (because changes in only the dark granules could be correlated with mechanical state: see below). The size of the dark and light granules did not differ significantly between the three mechanical states, and there was no significant difference between the quantity of light granules. However, dark granules were much more numerous in standard than in compliant and stiff CDLs, implying that dark granules are involved in the standard→compliant and standard→stiff events. How can the dark granules have a role in both antagonistic processes? An unusual feature of the JLCs of the CDL is that only one cell-type has been identified [22]. Most other MCTs possess at least two JLC types distinguishable by the size and/or shape of their granules. It is believed that these are also functionally distinct, perhaps representing separate ‘stiffener’ and ‘de-stiffener’ cells [2].

To explain these results we therefore hypothesize that:

1. There are two populations of CDL granules (each comprising mature and immature stages), which are functionally distinct (one is involved in the standard→stiff shift and the other in the standard→compliant shift), but not morphologically distinct. These may be present in separate, but morphologically indistinguishable, cell-types or in a single cell-type.
2. Only mature (i.e. ‘dark’) granules are involved in their respective processes, and as a consequence of this involvement they are depleted by an as yet unknown mechanism. They do not, through a decrease in the electron density of their contents, become light granules, because there is no corresponding increase in the number of light granules. After examination of many electron micrographs, we have found no evidence that JLC granules undergo exocytosis and we can only surmise that the transport of their contents to the extracellular environment and the recycling of their membranes are achieved by particularly rapid and efficient processes.

Living systems in nature are frequently multifunctional and dynamic, providing a source of inspiration for the design and synthesis of new classes of materials that have potentially a wide range of medical and non-medical applications [39], [40]. A priority in the biomedical field is the development of biomaterials that mimic ECM microenvironments and that allow a dynamic and two-way dialogue between the microenvironment and the cells, whilst also degrading at a rate similar to that at which the new tissue is being formed. The use of echinoderms as an animal model creates a unique opportunity to find new concepts for the development of a biomaterial that favours tissue regeneration, since their ECM, which closely resembles that of mammals, and particularly MCT, is present at anatomical sites at which there is a strong regenerative capacity [41]. In the present work, the sea-urchin CDL was used as a model MCT that has the advantages of being easily accessible and having a typical MCT organization uncomplicated by the incorporation of skeletal components. Although the molecular mechanism of mutability is understood incompletely, the concept is sufficiently appealing to deserve further consideration by biomaterials scientists.
